# A Virulent *Trueperella pyogenes* Isolate, Which Causes Severe Bronchoconstriction in Porcine Precision-Cut Lung Slices

**DOI:** 10.3389/fvets.2021.824349

**Published:** 2022-01-31

**Authors:** Lei Qin, Fandan Meng, Haijuan He, Yong-Bo Yang, Gang Wang, Yan-Dong Tang, Mingxia Sun, Wenlong Zhang, Xuehui Cai, Shujie Wang

**Affiliations:** ^1^National Key Laboratory of Veterinary Biotechnology, Harbin Veterinary Research Institute, Chinese Academy of Agricultural Sciences, Harbin, China; ^2^College of Veterinary Medicine, Northeast Agricultural University, Harbin, China; ^3^Institute of Animal Husbandry, Heilongjiang Academy of Agriculture Sciences, Harbin, China

**Keywords:** *Trueperella pyogenes*, porcine precision-cut lung slices, virulent, bronchoconstriction, infection

## Abstract

*Trueperella pyogenes* causes disease in cattle, sheep, goats and swine, and is involved occasionally in human disease worldwide. Most reports implicating *T. pyogenes* have been associated with clinical cases, whereas no report has focused on pathogenicity of *T. pyogenes* in mouse models or precision-cut lung slice (PCLS) cultures from swine. Here, we isolated and identified a virulent, β-hemolytic, multidrug-resistant *T. pyogenes* strain named 20121, which harbors the virulence marker genes *fimA, fimE, nanH, nanP* and *plo*. It was found to be highly resistant to erythromycin, azithromycin and medemycin. Strain 20121 was pathogenic in mouse infection models, displaying pulmonary congestion and inflammatory cell infiltration, partial degeneration in epithelial cells of the tracheal and bronchiolar mucosa, a small amount of inflammatory cell infiltration in the submucosa, and bacteria (>10^4^ CFU/g) in the lung. Importantly, we used *T. pyogenes* 20121 to infect porcine precision-cut lung slices (PCLS) cultures for the first time, where it caused severe bronchoconstriction. Furthermore, dexamethasone showed its ability to relieve bronchoconstriction in PCLS caused by *T. pyogenes* 20121, highlighting dexamethasone may assist antibiotic treatment for clinical *T. pyogenes* infection. This is the first report of *T. pyogenes* used to infect and cause bronchoconstriction in porcine PCLS. Our results suggest that porcine PCLS cultures as a valuable 3D organ model for the study of *T. pyogenes* infection and treatment *in vitro*.

## Introduction

*Trueperella pyogenes* is a Gram-positive, non-motile, non-spore-forming, short, rod- to coccobacillus-shaped bacterium that occurs singly, in pairs or in clusters. *T. pyogenes* was previously called *Corynebacterium pyogenes, Actinomyces pyogenes* and *Arcanobacterium pyogenes*, in chronological order (1, 2). In 2011, according to Yassin et al., *Arcanobacterium pyogenes* was renamed as *T. pyogenes* based on phylogenetic and chemotaxonomic observations (2).

*T. pyogenes* expresses several established and putative virulence factors. To date, known virulence factors mainly include exotoxin pyolysin (PLO), and others promote adhesion factors such as fimbriae (Fim), neuraminidase (NanH, NanP) and collagen-binding protein (CbpA) (3, 4). The cytolysin PLO is considered to be a major virulence factor, associated with cell damage induced by *T. pyogenes* infection (5). Adhesion factors may be associated with mucosal adherence and colonization of host tissues, thereby contributing to the pathogenicity of *T. pyogenes* (3).

The use of precision-cut lung slices (PCLS) in three-dimensional (3D) organ models is becoming an area of intensive research due to its close similarity to the host environment (6, 7). There are many advantages in the use of PCLS, including isolated tracheal rings and bronchial rings, and the contraction of airway (7). PCLS have been commonly used in the study of viral infections (8), but there have been few studies on bacterial pathogens (9). Porcine PCLS have been reported for the study of *Streptococcus suis* (10, 11), but never for *T. pyogenes*.

Here, we used a porcine PCLS model to study a *T. pyogenes* isolate, showing that infection of lung tissues by strain 20121 (isolated and characterized from lung tissues of clinically diseased pigs) resulted in severe bronchoconstriction. This study demonstrates that porcine PCLS is a suitable 3D organ model for the study of pathogenicity of *T. pyogenes* in *vitro*.

## Materials and Methods

### Ethics Statement

All animal experiments were conducted in accordance with the Guide for the Care and Use of Laboratory Animals of the Ministry of Science and Technology of the People's Republic of China. Mouse infection experiments (approval number 210119-02) were carried out in the animal biosafety level 2 facilities under the supervision of the Committee on the Ethics of Animal Experiments of the Harbin Veterinary Research Institute of the Chinese Academy of Agricultural Sciences (CAAS) and the Animal Ethics Committee of Heilongjiang Province, China.

### Bacterial Strains

The novel *T. pyogenes* strain 20121 was isolated from the lungs of two sick nursery pigs with pneumonia and severe abdominal effusion from a pig farm in Heilongjiang Province of China in 2020. The lung samples were cultured on Columbia-based blood agar media (ThermoFisher Scientific, Beijing, China) for 36 h at 37 °C. The isolated colonies were cultured for 36–48 h in tryptic soy agar (TSA, Difco, Loveton Circle Sparks, MD, USA) plates supplemented with 5% fetal bovine serum (FBS, CLARK, USA) at 37 °C and inoculated in tryptic soy broth (TSB, Difco, Loveton Circle Sparks, MD, USA) supplemented with 5% FBS for extraction of genomic DNA.

The isolate 20121 in TSB was observed after rapid Gram stain (AOBOX, Beijing, China) following standard procedure using an optical microscope (Primo Star, ZEISS). Furthermore, the isolate was prepared with standard electron microscopy procedures and the morphological structure was observed using a Hitachi H-7650 transmission electron microscope.

### PCR Detection

Genomic DNA of *T. pyogenes* 20121 was extracted by a Bacterial DNA Extraction Kit (Tiangen, Beiing, China) following the manufacturer's instructions. *T. pyogenes* was positively identified by detecting the 16S rRNA (12) products through PCR. The virulence factors of *T. pyogenes* 20121 were detected by PCR (13), including hemolysin (pyolysin, PLO), collagen-binding protein (CbpA), neuraminidase (NanH and NanP), and fimbriae (FimA, FimC, FimE and FimG). The primer sequences and reaction conditions are listed in Table 1. PCR products were subsequently analyzed by loading to 1% agarose gels with 2000 bp marker (Tiangen, Beiing, China) and purified with a Gel Extraction Kit (OMEGA, New York, USA). Then, the PCR products were cloned into a pMD18-T vector (Takara, Dalian, China) and positive clones were sequenced by Comate Bioscience Company Ltd.

**Table 1:** 16S RNA and the virulence factor genes used in this study.

**Target gene**	**Primers (5′-3′)**	**Size of target amplicon (bp)**
*16S rRNA*	5′-AGAGTTTGATCCTGGCTCAG-3′	1,465
	5′- TACGGCTACCTTGTTACGACTT-3′	
*cbpA*	5′-GCAGGGTTGGTGAAAGAGTTTACT-3′	124
	5′-GCTTGATATAACCTTCAGAATTTGCA-3′	
*fimA*	5′-CACTACGCTCACCATTCACAAG-3′	605
	5′-GCTGTAATCCGCTTTGTCTGTG-3′	
*fimC*	5′-TGTCGAAGGTGACGTTCTTCG-3′	843
	5′-CAAGGTCACCGAGACTGCTGG-3′	
*fimE*	5′-GCCCAGGACCGAGAGCGAGGGC-3′	775
	5′-GCCTTCACAAATAACAGCAACC-3′	
*fimG*	5′-ACGCTTCAGAAGGTCACCAGG-3′	929
	5′-ATCTTGATCTGCCCCCATGCG-3′	
*nan-H*	5′-CGCTAGTGCTGTAGCGTTGTTAAGT-3′	781
	5′-CCGAGGAGTTTTGACTGACTTTGT-3′	
*nan-P^1^*	5′-ATGATGAGCGCCCGCGTGGGCGGGGGTA-3′	2,275
	5′-TAACCGAGTTCGCCGCAAGCGCTAGTTT-3′	
*plo*	5′-GGCCCGAATGTCACCGC-3′	270
	5′-AACTCCGCCTCTAGCGC-3′	

*1, Designed for use in current study*.

### Antibiotic Susceptibility Test

The susceptibility of *T. pyogenes* 20121 to different antibiotics was determined by the drug sensitive paper disc diffusion method, according to the Clinical and Laboratory Standards Institute (CLSI) guidelines (2016). The following antimicrobials were used: cefazolin, nitrofurantoin, erythromycin, chloramphenicol, kanamycin, ampicillin, ceftazidime, clarithromycin, meropenem, azithromycin, trimethoprim, medemycin, spiramycin, fosfomycin, ceftriaxone, cefoxitin, streptomycin, tetracycline, ciprofloxacin and vancomycin (Tianhe, Hangzhou, China). The quality control strain *Streptococcus pneumoniae* (ATCC49619) was stored in our lab (12).

### Phylogenetic Analysis

The similarities between the nucleotide sequences recovered from isolate 20121 and the reference *T. pyogenes* sequence published in GenBank were aligned using BLAST online search tool (http://blast.ncbi.nlm.nih.gov). Phylogenetic trees of genes *16S rRNA* and *plo* sequences were constructed with MEGA V 7.0 using the neighbor-joining method and a bootstrap validation with 1,000 replications. Branches corresponding to partitions reproduced in <50% bootstrap replicates were collapsed and shown above the branches. Data from the *T. pyogenes 16S rRNA* and *plo* genes used for phylogenetic trees are listed in Tables 2, 3.

**Table 2:** *Trueperella pyogenes 16S rRNA* genes referenced in this study.

**Strain**	**Isolation source**	**Host**	**Country**	**Accession no**.
NIAH 13535	Abscess in leg of swine	Sus scrofa	Japan	LC500012
NIAH 13534	The lung of diseased swine	Sus scrofa	Japan	LC500011
24398	Vaginal discharge	Okapia johnstoni	Germany	MN946520
HC-H 13-2	Uterine secretions	Cattle	China, Liaoning	EU268191
TP6375	Uterus with metritis	Dairy cow	USA	CP007519
DAT1453	Ileum	Sus scrofa	Japan	LC500013
Bu8-2B2	Abortion material	Bubalus bubalis	India	MG461533
H9	Intrauterine fluid	Buffalo	China, Guangxi	KC894522
DTK434	Abscess in the brain of goat	Capra hircus	Japan	LC500006
DTK435	The lung of diseased sheep	Ovis aries	Japan	LC500004
G	Lung	Calf	China, Hebei	KP159746
S 1276/1/18	Lung	Lynx	Germany	MN135984
171003246	Kidney	Python regius	Germany	MN712476
141010414	Brain abscess	Capreolus capreolus	Germany	KX815984
TP8	Pus	forest musk deer	China, Sichuan	CP007003
TP2	Knee joint	Bovine	China, Jilin	CP033903
XJXBMY-11-4NF	Lung with pneumonia	Bos Taurus	China, Xinjiang	JQ975936
M29	Pus samples	Forest musk deer	China, Sichuan	JN578115
scnu001	Pus	Goat	South Korea	MT775813
11-07-D-03394	Facial abscess	gray slender lorises	Germany	HG530069
TP3	Lung	Swine	China, Jilin	CP033904
Truep25	Pus	Swine	Brazil	KJ930040
AUVF-TRU_19	Vaginal swap	Cattle	Turkey	MN907639
FL-1	Pus	Goat	China, Chongqing	KX462008
XJALT-127-2YF1	Lung	Goat	China, Xinjiang	JX975440
HJ-4	Pus	Dairy cattle	China, Hei Longjiang	GU372928
TP-2849	Lung	Swine	China, Jilin	CP029004
2012CQ-ZSH	Goat lung tissue	Capra aegagrus	China, Chongqing	CP012649
15A0121	Abortion (placenta)	Bos taurus	Switzerland	CP063213
19OD0592	Lung	Sus scrofa	Switzerland	CP063212
SCDR 1	Patient	Homo sapiens	Saudi Arabia	CP034038
jx18	Lung	Swine	China, Jiangxi	CP050810
TP4	Lung	Swine	China, Jilin	CP033905
TP1	Lung	Bovine	China, Jilin	CP033902
Arash114	Uterine secretions	Water Buffalo	Iran: Tehran	CP028833
FC3480	Patient	Homo sapiens	China	MK611773
nck254a04c1	Skin, antecubital fossa	Homo sapiens	USA	KF098604
S350	Cord blood unit	Bovine	Portugal	KR232876
P504064-19-1	Abortion material	Pig	United Kingdom	MW332266
IMMIB L-1653	Abortus	Sus scrofa	Germany	HE575404
JCM 14813	Sow placenta after abortion	Sow	Japan	LC500014
Murakami	Placenta of an aborted sow	Sow	Japan: Chiba	NR 041607

**Table 3:** *Trueperella pyogenes pyolysin* genes referenced in this study.

**Strain**	**Isolation source**	**Host**	**Country**	**Accession no**.
24398	Vaginal discharge	Okapia johnstoni	Germany	MN956806
S 1276/1/18	Lung	Lynx	Germany	MN163264
171003246	Kidney	Python regius	Germany	MN741110
DTK435	The lung of diseased sheep	Ovis aries	Japan	LC500001
DTK434	Abscess in the brain of goat	Capra hircus	Japan	LC500002
FMV13	Uterus	Bovine	Portugal	KJ150328
HJ-3	Pus	Dairy cattle	China, Helongjiang	HQ637573
TP2	Knee joint	Bovine	China, Jilin	CP033903
jx18	Lung	Swine	China, Jiangxi	CP050810
NIAH 13534	The lung of diseased swine	Sus scrofa	Japan	LC500003
2012CQ-ZSH	Goat lung tissue	Capra aegagrus hircus	China, Chongqing	CP012649
TP6375	Uterus with metritis	Dairy cow	USA	CP007519
Arash114	Uterine secretions	Water Buffalo	Iran: Tehran	CP028833
TP8	Pus	Forest musk deer	China, Sichuan	CP007003

### Experimental Mouse Infection

To investigate the virulence of *T. pyogenes* 20121, a mouse survival experiment was carried out. Briefly, 35 six-week-old (18–20 g) female C57BL/6 mice (Changsheng Biotechnology, Liaoning, China) were randomly divided into 5 groups including a non-infection control. The mice were challenged intraperitoneally (i.p.) with 0.2 ml bacterial suspension containing strain 20121 (2 × 10^6^, 2 × 10^7^, 2 × 10^8^ and 2 × 10^9^ CFU) or sterile TSB. Each group contained 6 mice except for the group receiving 2 × 10^6^ CFU, which had 11 mice. Clinical symptoms and mortality were recorded for 14 days, during which any mice exhibiting extreme lethargy were considered moribund and were humanely euthanized. Samples of blood and organs (lung, trachea, heart, liver, ileum, duodenum, spleen and thymus) of infected mice were collected and observed for gross pathological changes. Organ/body weight (g/g) × 100% was calculated and organ samples were fixed immediately in 3.7% formaldehyde (Amresco, Fountain Parkway, USA) for histopathological examination. 5 μl of anticoagulated blood and lung samples were serially diluted in PBS, and plated on blood agar medium for 36 h, and the bacteria in blood and lung samples were quantified by colony counting. 60 μl of anticoagulated blood was tested by a ProCyte Dx automatic blood cell analyzer (IDEXX, USA), determining white blood cell count (WBC), reticulocyte (RET), platelet (PLT), neutrophilic granulocyte percentage (NEUT%), lymphatic number percentage (LYMP%), monocyte percentage (MONO%), eosinophil percentage (EO%) and hematocrit percentage (HCT%).

### *T. pyogenes* 20121 Infection of Porcine PCLS

Fresh lung tissue was obtained after euthanasia from three 3-month-old SPF pigs from Harbin Veterinary Research Institute. The cranial, middle, and intermediate lobes were filled with low-melting agarose (Promega, Madison WI, USA) along the bronchus gently as described previously (14). The filling tissue were covered by ice until the agarose became solidified, then the tissue was stamped out as cylindrical portions with 8-mm tissue coring tool and precision slices were further prepared by a Krumdieck tissue slicer (model MD6000-01; TSE Systems). The PCLS were carefully picked up into 24-well plat (one slice/well) maintained with 1ml fresh medium for additional cultivation for 24 h. Then the airway epithelial cells with 100% ciliary activity were divided randomly into three groups. PCLS were washed 3 times with PBS, then inoculated with *T. pyogenes* 20121 (8 × 10^4^ CFU or 8 × 10^5^ CFU per slice) in a humidified atmosphere containing 5% CO_2_ at 37 °C, mock-infected slices were used as control. Infected slices were washed three times with PBS to remove non-attached bacteria at 4 h post-inoculation (hpi), and 1 ml fresh medium (1640, Gibco, Beijing, China) was added for further cultivation.

*T. pyogenes* 20121-induced bronchoconstriction was detected by imaging and measurement of bronchial cavity area. Initial bronchial cavity area was calculated for each PCLS by imaging at 0 hpi by inverted light microscope (EVOS FL Auto, ThermoFisher Scientific). To quantify the relative bronchoconstriction of the lung tissue, bronchial cavity positions in each infected and mock-infected PCLS were imaged at 4 and 24 hpi. Bronchial cavity areas were measured and calculated using ImageJ/Fiji and the results were presented as bronchial contraction percentage (BCP) using the formula, BCP = [reduced bronchial cavity area / initial bronchial cavity area] × 100%. Treatments were carried out in triplicate and experiments were repeated at least three times.

In order to evaluate the drug intervention effect on bronchoconstriction induced by *T. pyogenes* 20121, several bronchodilators were selected and applied in this study. Dexamethasone (785 μg/ml in final concentration), atropine (300 μg/ml in final concentration), aminophylline (420.43 μg/ml in final concentration), α-Terpineol (168 μg/ml in final concentration) or salbutamol (250 μg/ml in final concentration) were added at the time of inoculation with strain 20121 (8 × 10^5^ CFU), and then non-attached bacteria were removed at 4 hpi followed by adding fresh medium containing the appropriate drugs.

### Statistical Analyses

Numerical data were expressed as the mean ± standard deviation (SD). Statistical analysis of the results was performed with one- or two-way ANOVA using GraphPad Prism version 9.00 (GraphPad, San Diego, CA, USA). Statistical significance was evaluated based on Bonferroni post-tests, and *p* < 0.05 was considered statistically significant.

## Results

### Morphological Evaluation and Virulence Factors of *T. pyogenes* 20121

**Figure 1:**
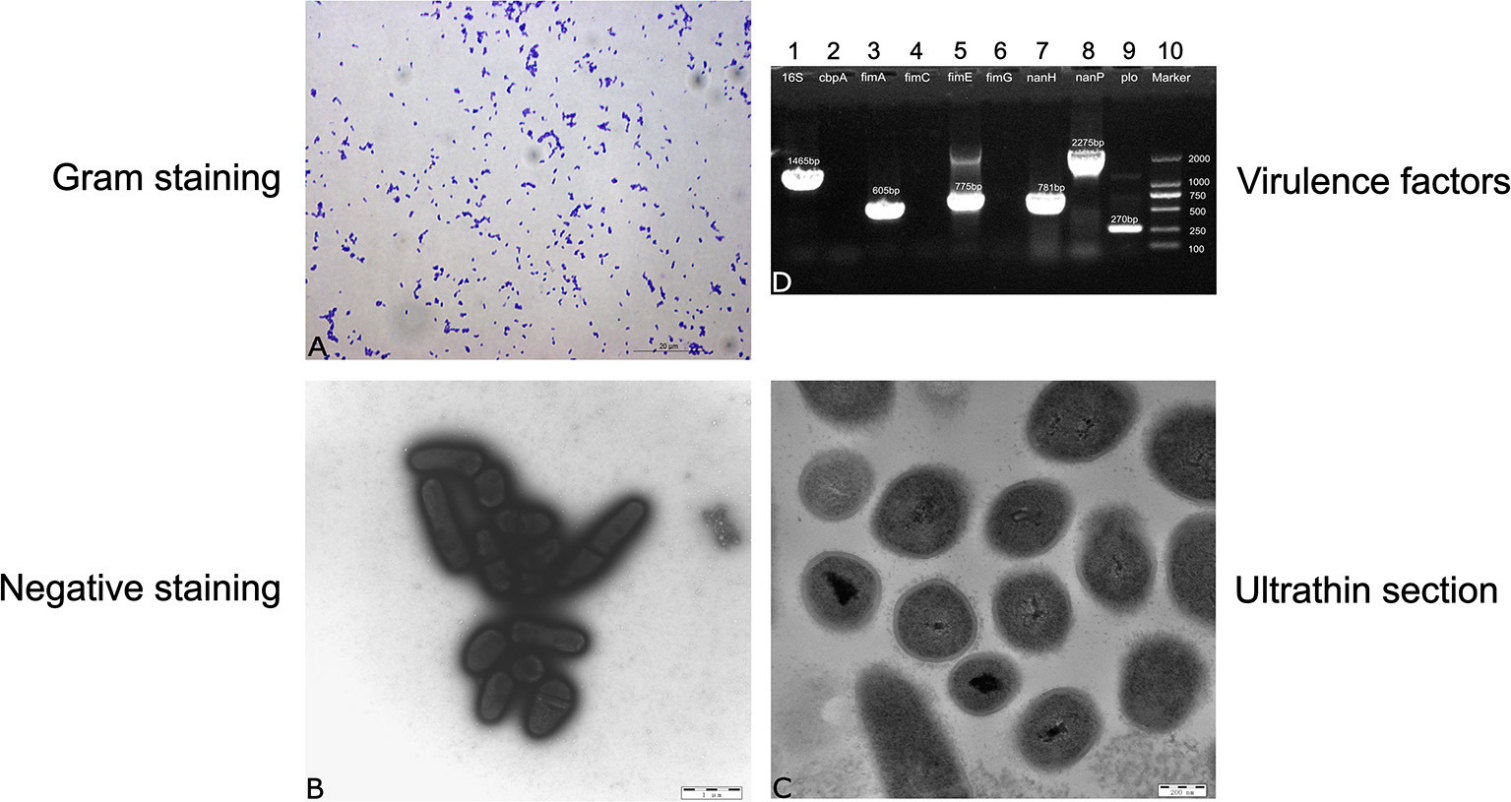
Morphological evaluation and screening of genes encoding virulence factors. **(A)** Gram staining for strain 20121, **(B)** negative staining for strain 20121 on transmission electron microscope **(C)** ultrathin section for strain 20121 on transmission electron microscope, **(D)** PCR analysis of virulence factors genes, 1: *16S-rRNA*, 2: *cbpA*, 3: *fimA*, 4: *fimC*, 5: *fimE*, 6: *fimG*, 7: *nan-H*, 8: *nan-P*, 9: *plo*, 10: Marker.

Strain 20121 was isolated from two lungs of two sick pigs from Heilongjiang Province of China in December 2020. The isolate shows a narrow zone of β-hemolysis with 0.1-mm hemolytic rings and formed small, white, wet, smooth, and glossy colonies on Columbia blood agar media after culturing for 48 h. It is a Gram-positive coccobacilli or short coryneform occuring in single or pairs (Figure 1A). Electron micrographs of negative staining showed that the isolate were corynebacteria (Figure 1B). Electron micrographs of ultrathin sections showed the strain was most likely encapsulated with 10–25 nm cell wall thickness (Figure 1C). *16S rRNA* sequencing indicated that the isolate was *T. pyogenes*, which harbored virulence factor genes *plo, fimA, fimE, nanH* and *nanP*.

### Sequence Comparison and Phylogenetic Analysis of Strain 20121

Genes *16S rRNA, plo, fimA, fimE, nanH, nanP* of strain 20121 were sequenced and compared to *T. pyogenes* strains in NCBI GenBank. *16S rRNA* sequence of strain 20121 shared >94% nucleotide identity with sequences in GenBank (Figure 1D). In addition, the virulence factors *plo* (accession MZ189360), *fimA* (accession MZ189359), *fimE* (accession MZ579543) and *nanP* (accession MZ579545) were highly similar, sharing >96, >98, >96, and >97% nucleotide identity, respectively, with sequences in GenBank. The *nanH* sequence (accession MZ579544) from strain 20121 was less similar, with only 81–89% nucleotide identity with homologous sequences from *T. pyogenes* strains in GenBank.

**Figure 2:**
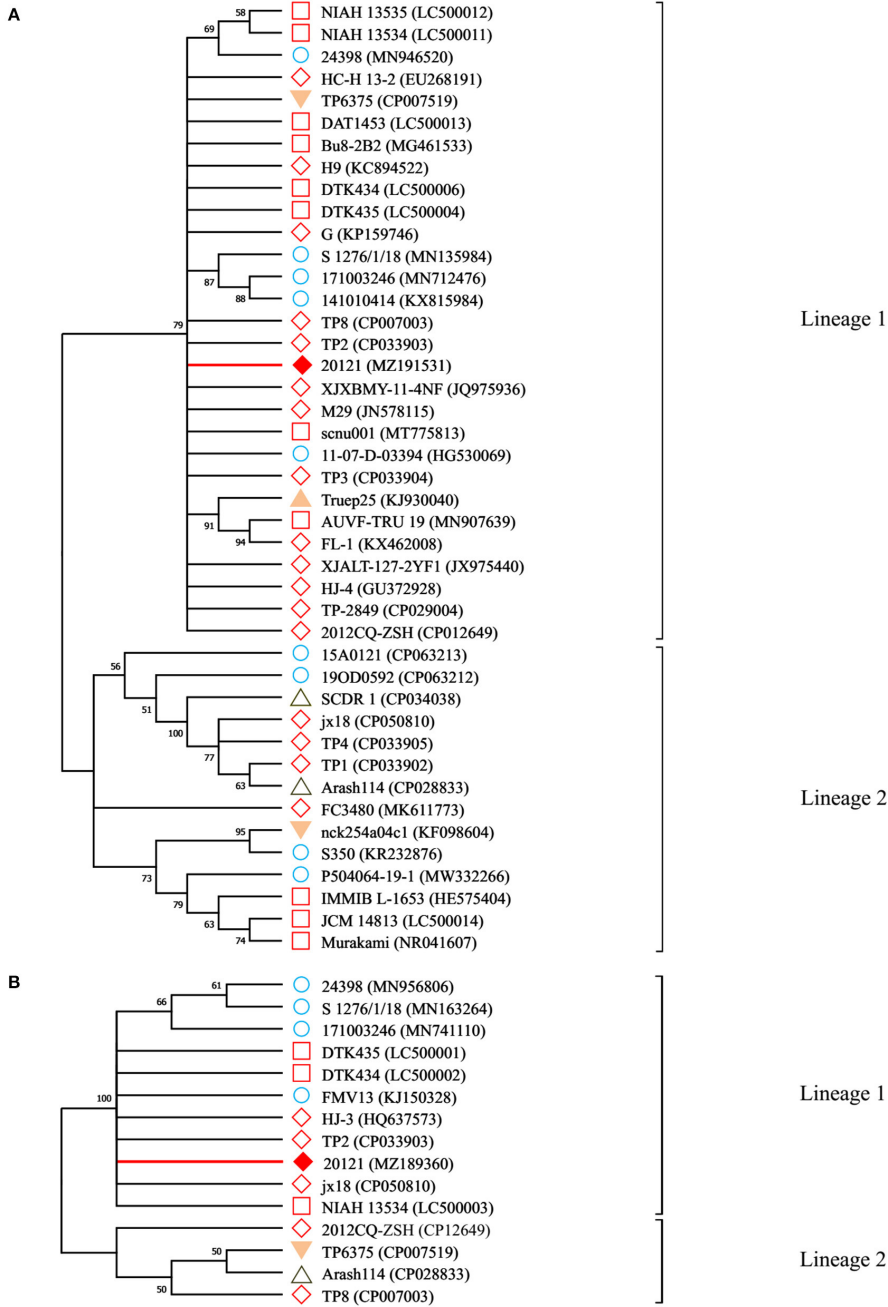
Neighbor-joining phylogenetic trees for *16S RNA* and *plo* genes. **A**: the phylogenetic trees for 16S RNA gene; **B**: the phylogenetic trees for plo genes. Branches corresponding to partitions reproduced in <50% bootstrap replicates are collapsed. The percentage of replicate trees in which the associated taxa clustered together in the bootstrap test (1,000 replicates) are shown above the branches. ♦ denotes the genes sequenced in this study; ♢ indicates the reference sequences isolated from China; while □ indicates sequences isolated from the rest of Asia; ◦ are sequences isolated from European countries; △ indicates isolated reports from the Middle Eastern countries, ▴ was from a South American country and ▾ denotes isolates from North American countries.

To analyze the evolutionary relationship between strain 20121 and other *T. pyogenes* strains, we constructed phylogenetic trees based on the *16S rRNA* Figure 2A and *plo* Figure 2B. As shown in Figure 2, strain 20121 is located within lineage 1 of the trees based on the *16S rRNA* and *plo* genes, in the same lineage as most Chinese and other Asian isolates, and some European ones. The sequences within lineage 1 shared >99 and >98% nucleotide identity, respectively.

### Antimicrobial Susceptibility Profiles of Strain 20121

In order to determine the drug resistance profile and thus the best way to potentially treat sick animals, we tested strain 20121 for susceptibility to cefazolin, nitrofurantoin, erythromycin, chloramphenicol, kanamycin, ampicillin, ceftazidime, clarithromycin, meropenem, azithromycin, trimethoprim, medemycin, spiramycin, fosfomycin, ceftriaxone, cefoxitin, streptomycin, tetracycline, ciprofloxacin and vancomycin. The test strain was highly resistant to erythromycin, azithromycin and medemycin, with growth exclusion diameters of 6 mm, 10 mm, and 8 mm, respectively, while it was sensitive to the other antibiotics.

### Virulence Evaluation of 20121 Isolate in Mice

**Figure 3:**
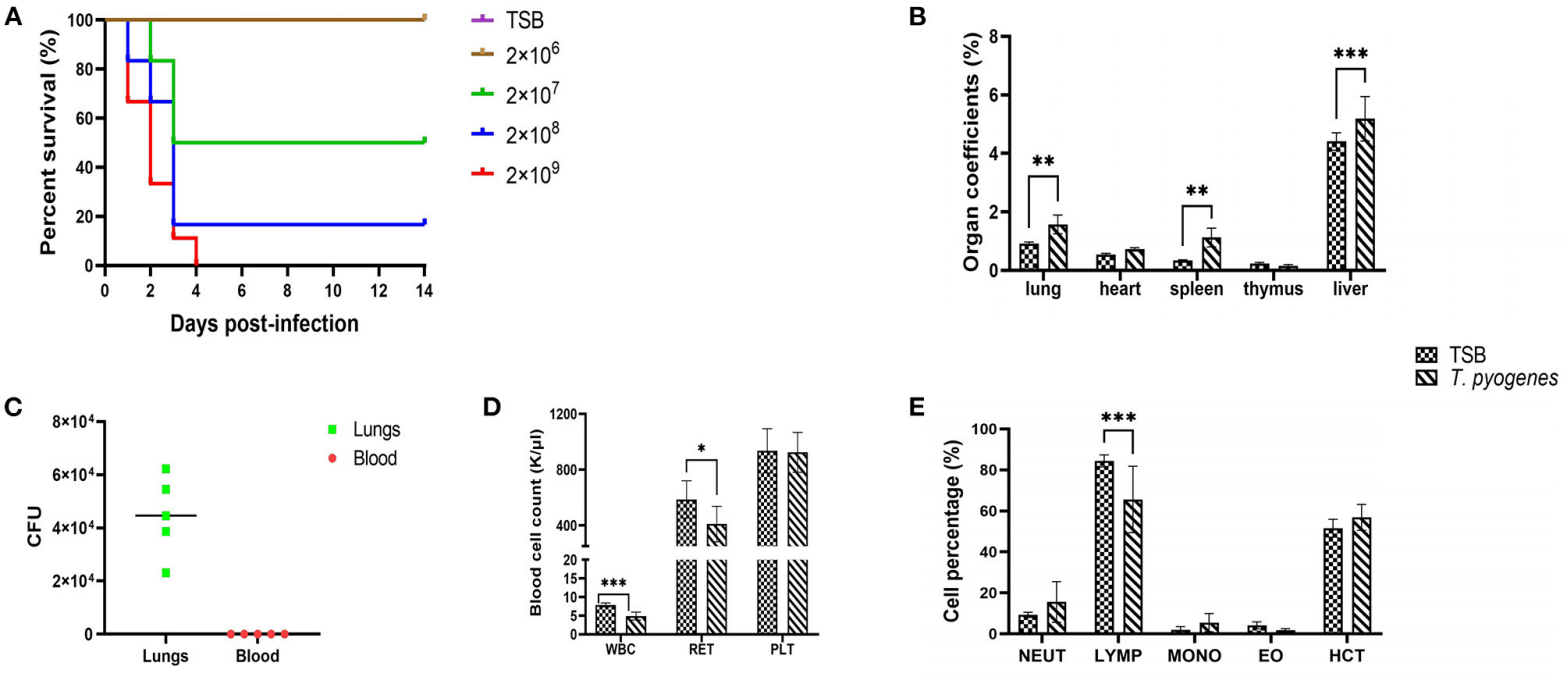
Virulence of *T. pyogenes* strain 20121 in a mouse model. Mice were challenged intraperitoneally with the strain 20121, as described in the Experimental Procedure. **(A)** Survival percentage of mice inoculated with strain 20121 or tryptic soy broth (TSB; control). **(B)** Organ coefficients after 20121 infection at 2 dpi. **(C)** Bacterial loads in lung (CFU/g of tissue) and blood (CFU/ml). **(D)** White blood cell (WBC), reticulocyte (RET) and platelet (PLT) count after *T. pyogenes* infection. **(E)** Percentage of neutrophilic granulocyte (NEUT), lymphatic number (LYMP), monocyte (MONO), eosinophil (EO), hematocrit (HCT) after *T. pyogenes* infection. Results are expressed as means ± S.D., and significance was determined using two-way ANOVA and the Sidak multiple-comparison test. *p < 0.05; **p < 0.01; ***p < 0.001.

To evaluate the virulence of strain 20121 *in vivo*, we injected six-week-old C57BL/6 mice and tracked their survival and clinical signs. The mice infected with 20121 exhibited depression, trembling, exuberant periocular secretion and fecal adhesions at the anus after injecting 20121, and the clinical signs were bacteria dose-dependent. In the higher dose groups, all of the mice receiving 2 × 10^9^ CFU and most (5/6) of the mice who received 2 × 10^8^ CFU died. The survival percentage of the 2 × 10^7^ CFU and 2 × 10^6^ CFU groups were 50% and 100%, respectively (Figure 3A).

On 2 dpi, five mice in the 2 × 10^6^ CFU group and three control mice were sacrificed, and the main gross lesions in various organs were visualized. Organ-body weight ratios for lung, heart, spleen, thymus and liver were calculated. As shown in Figure 3B, all coefficients of the lungs, spleen and liver were significantly increased (*p* < 0.01). In addition, bacteria were detected in the lungs of infected mice, revealing higher levels (>10^4^ CFU/g tissue) on 2 dpi (Figure 3C). No bacteremia was observed at 2 dpi as determined by colony count after plating 5 μl blood on blood agar media (Figure 3C).

### *T. pyogenes* 20121 Infection Induces Abnormal Hematological Parameters

We used an automatic blood cell analyzer to detect WBC, RET, PLT, NEUT%, LYMP%, MONO%, EO% and HCT% at 2 dpi. *T. pyogenes* 20121 infection altered the expression of hematological parameters which can influence immunity. As shown in Figure 3D, the blood levels of WBC decreased significantly (*p* < 0.001) and RET decreased slightly (*p* < 0.05) at 2 dpi, and no significant differences in the levels of blood of PLT. Meanwhile, LYMP% in blood of infected mice had a significant decrease (*p* < 0.001), with 18.8% less than control mice at 2 dpi, but no significant differences in NEUT, MONO, EO, and HCT% in blood (Figure 3E).

### *T. pyogenes* 20121 Causes Histopathological Lesions in a Mouse Model

**Figure 4:**
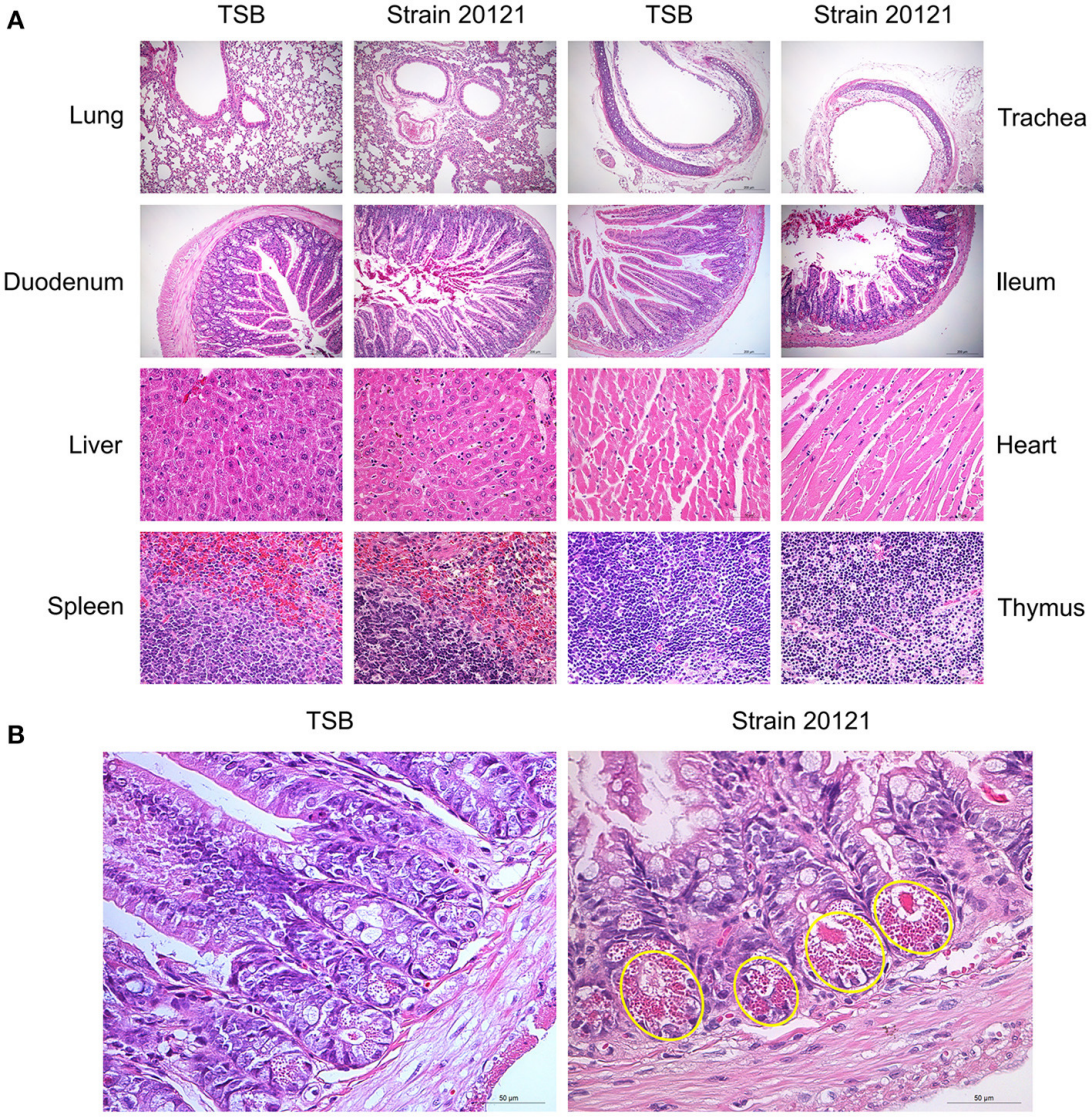
Organ histopathology in mice infected with *T. pyogenes*. **(A)** Mice were infected with 2 × 10^6^ CFU of *T. pyogenes* strain 20121 or inoculated with tryptic soy broth (TSB) as control. Lung, trachea, duodenum, ileum (scale bars = 200 μm), liver, heart, spleen and thymus (scale bars = 50 μm) were collected on 2 dpi for observation of histopathological lesions. **(B)** Ileum at 2 dpi, showing degeneration of mucosal epithelial cells, necrosis and sloughing, and Paneth cell degeneration (yellow oval); scale bars = 50 μm.

Histopathological lesions were determined in mice that were euthanized on 2 dpi (Figure 4A). In 20121-infected mice, the main histopathological lesions observed in the lungs were pulmonary congestion and inflammatory cell infiltration, moderately broadened alveolar diaphragm, partial degeneration in the epithelial cells of the tracheal and bronchial mucosa, and a small amount of inflammatory cell infiltration in the submucosa. In infected livers, extensive degeneration of hepatocytes, partial necrosis, and small focal clusters of Kupffer cells could be seen locally. Extensive necrosis and nuclear pyknosis of thymocytes and infiltration of inflammatory cells into the lamina propria of the chorionic mucosa were seen, along with massive necrosis of lymphocytes in the submucosal lymphatic tissue of the duodenum. A small amount of mucosal epithelial cells degenerated, necrosed and fell off, and Paneth cell degeneration was observed in the ileum (Figure 4B). The main histopathological lesions observed in the spleen were white pulp atrophy and lymphopenia. However, there was no significant changes in the infected heart tissue.

### *T. pyogenes* 20121 Induces Severe Bronchoconstriction in PCLS Cultures

**Figure 5:**
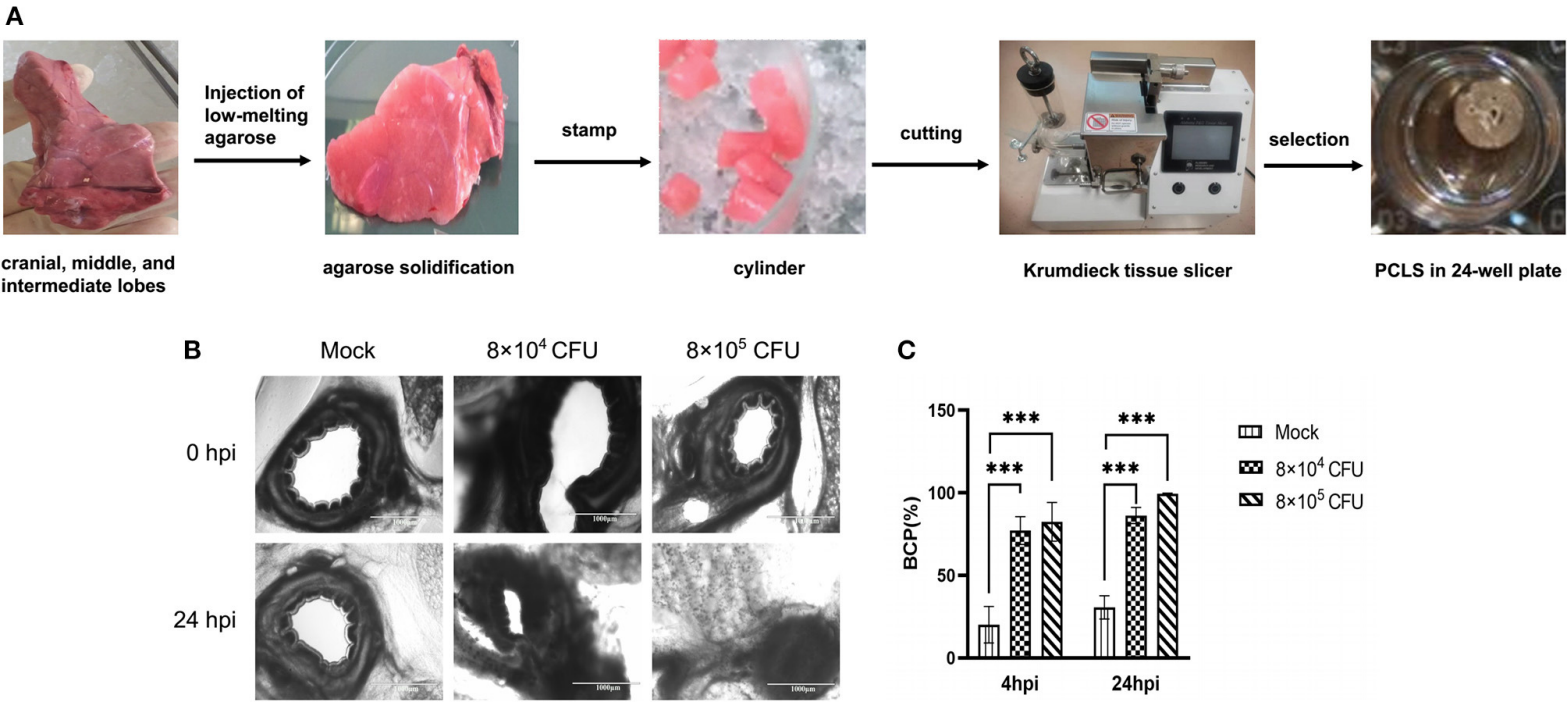
*T. pyogenes* 20121 induced bronchoconstriction in porcine PCLS cultures. **(A)** The PCLS preparation process; **(B)** Light microscope imaging of bronchial cavities in infected (8 × 10^4^ or 8 × 10^5^ CFU) and mock PCLS cultures at 4 and 24 hpi; **(C)** Bronchial cavity area was measured and calculated as bronchial contraction percentage (BCP). Results are expressed as means ± S.D., and significance was determined using two-way ANOVA and the Tukey multiple-comparison test. ***p < 0.001.

It has been reported that bacterial infection in the respiratory tract may contribute to development of bronchospasm and the progression of chronic obstructive pulmonary disease (COPD) (15). To further evaluate the damage to pig lungs induced by *T. pyogenes* 20121, we prepared the porcine PCLS and the preparation process was showed in Figure 5A. Then different doses were used to inoculate PCLS for 4 or 24 h. Interestingly, we found that *T. pyogenes* infection was able to cause obvious bronchoconstriction on PCLS (Figure 5B). A slightly constriction can be observed in bronchus of infected PCLS at 2 hpi. The bronchial cavities of infected PCLS showed obvious reduction in area relative to control PCLS at 4 hpi; the BCP of the 8 × 10^4^ CFU group and 8 × 10^5^ CFU group were nearly 77.11 and 82.44% (Figure 5C), respectively. At 24 hpi, the bronchial cavity area remained nearly 14% (BCP: 86.31%) in the 8 × 10^4^ CFU group, and the area of bronchial cavity almost disappeared completely in 8 × 10^5^ CFU group (BCP: 99.53%) comparing with controls (Figure 5). The observed airway closure was persistent and could not be relieved by the replacement of fresh medium. The high dose infectious group induced a faster bronchoconstriction response, which indicates a dose-dependent capability of strain 20121 to trigger bronchoconstriction.

### Dexamethasone Relieves the Bronchoconstriction Caused by Strain 20121

**Figure 6:**
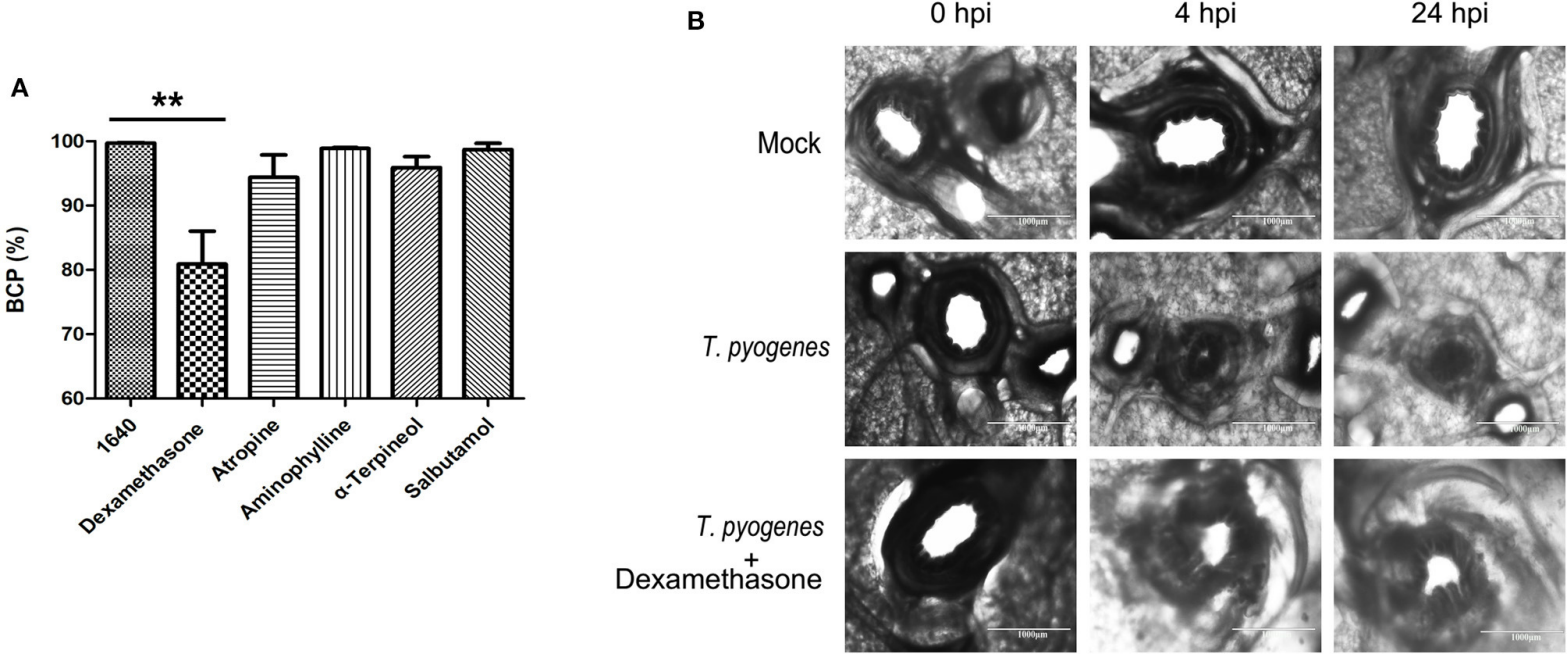
Therapeutic effect of bronchodilators on bronchoconstriction in infected PCLS cultures. **(A)** The BCP after five common bronchodilators were used to treat bronchoconstriction resulting from *T. pyogenes* infection. Results are expressed as means ± S.D., and significance was determined using one-way ANOVA and the Tukey multiple-comparison test. **p < 0.01. **(B)** Therapeutic effect of dexamethasone on bronchoconstriction in PCLS cultures infected with *T. pyogenes*.

Bronchodilating drugs can dilate the bronchi and bronchioles to improve lung ventilation and relieve wheezing. Five common bronchodilators (dexamethasone, atropine, aminophylline, α-Terpineol and salbutamol) were selected and applied to the PCLS infection model in order to improve bronchospasm and relieve bronchoconstriction caused by *T. pyogenes* infection. Among the five, our results showed that only dexamethasone relieved the bronchoconstriction caused by *T. pyogenes* 20121 at 4 hpi and 24 hpi, keeping 14.70 and 19.17% of bronchiole luminal area open, respectively, compared to the no-drug group (Figure 6). Thus, our results indicated that dexamethasone is a candidate for relief of bronchospasm in the treatment of *T. pyogenes* infection resulting in wheezing.

## Discussion

Although *T. pyogenes* has been especially linked to bovine mastitis, it is a well-known causative agent of diverse clinical presentations among domestic ruminants, pigs, companion animals and gray slender lorises (16). In domestic animals, there have been more bacterial species isolated from cattle and sheep than pigs over the last 10 years (17, 18). However, *T. pyogenes* infections in pigs have become an increasingly serious clinical problem on large-scale farms (19, 20). *T. pyogenes* had a high isolation rate in bacterial swine pneumonia in Jilin Province (21). Pneumonia in pigs is caused mainly by viruses and bacteria. In this study, we firstly detected common viruses on the lungs of sick pigs, and the results were all negative. Furthermore, we isolated bacteria and most of clones on the blood plate are *T. pyogenes*. In order to know the best way to potentially treat the sick animals, we conducted a drug sensitivity experiment and made recommendations to the affected pig farm that resulted in effective control of the disease.

Strain 20121 showed a β-hemolytic phenotype, implying that it could produce a hemolysin that dissolves red blood cells completely. This finding was further confirmed by detecting the virulence factor PLO in the strain. The genotype of virulence factors in strain 20121 was *plo/fimA*/*fimE*/*nanH*/*nanP*, genes which have also been identified in bovine mastitis, pneumonia and abscesses, as well as encephalitis in goats (22). This is the first report of the genotype *plo/fimA*/*fimE*/*nanH*/*nanP* identified in swine pneumonia. The sequences of *plo, fimA, fimE* and *nanP* were highly similar to sequences in GenBank, indicating that these genes are highly conserved in *T. pyogenes* species. However, the *nanH* sequence of strain 20121 was relatively less similar to other *nanH* sequences from GenBank, suggesting that it may be less conserved among *T. pyogenes* isolates.

This is the first report of *T. pyogenes* infection in a mouse model causing massive necrosis of lymphocytes in the submucosal lymphatic tissue of the duodenum. Intestinal Paneth cells are the “gatekeepers” of intestinal innate immunity, thus impacts to Paneth cells can cause intestinal inflammation and inflammatory bowel disease. Therefore, our results suggest that *T. pyogenes* infection of mice induces abnormal mucosal immune response, thereby downregulating the immune response. Also, the levels of WBC and LYMP% in blood decreased significantly at 2 dpi, indicating that *T. pyogenes* infection reduced the immune function in mice. What's more, there was extensive necrosis in thymocytes, suggesting that the central immune organ thymus was destroyed after *T. pyogenes* infection in mice. Altogether, we provided evidence to show that *T. pyogenes* decreases immune function in the early stages of infection, though much remains undetermined and warrants further investigation.

For the first time in swine PCLS cultures, we showed that infection of *T. pyogenes* induced severe bronchoconstriction and led to a narrowing of the luminal area of bronchioles, suggesting PCLS are a suitable 3D model for the study *T. pyogenes* infection. Narrowing of bronchioles would affect lung ventilation and cause severe airflow limitations, which may aggravate damage to the lungs and cause the host to die suddenly from asphyxiation. We speculate that an inflammatory response may responsible for contraction of airway smooth muscle induced by *T. pyogenes* infection. However, the mechanisms by which *T. pyogenes* causes bronchoconstriction remain unclear and warrants further study in the future.

PCLS cultures have been reported to preserve lung functions for around 10 days (23), during which the bronchioles can maintain reversible constriction after methacholine treatment (24). Thus, as a good model for analyzing the effect of drugs on lung tissue, PCLS cultures have been applied for toxicological and functional studies (25, 26). In the current study, we found that dexamethasone dilated airways and kept the bronchioles open, showing a protective effect for bronchoconstriction caused by *T. pyogenes* infection in porcine PCLS. Dexamethasone is a long-acting glucocorticoid that is widely used due to its anti-inflammatory and immunosuppressive properties (27). High-dose glucocorticoid intervention is used for the treatment of mild or severe asthma with sudden onset in human to improve alveolar ventilation while treating the underlying illness (28). With respect to treatment of *T. pyogenes* infection, we suggest that a combination therapy of dexamethasone and antibiotics should be taken into account in swine clinical practice. Moreover, PCLS can be used in the hunt for more effective therapeutic drugs for *T. pyogenes* infection.

In conclusion, we isolated and identified a multidrug-resistant *T. pyogenes* strain from the lungs of sick pigs. It was virulent in a mouse infection model and also led to heavy bronchoconstriction in porcine PCLS cultures. What's more, bronchodilators showed their ability to antagonize bronchoconstriction in the same cultures. In summary, our results highlight porcine PCLS cultures as a valuable 3D organ model for the study of *T. pyogenes* infection and treatment in *vitro*.

## Conclusion

A virulent, multidrug-resistant *T. pyogenes* strain induced severe bronchoconstriction in porcine PCLS, highlighting a valuable 3D organ model for the study of *T. pyogenes* infection *in vitro*.

## Data Availability Statement

The datasets presented in this study can be found in online repositories. The names of the repository/repositories and accession number(s) can be found below: https://www.ncbi.nlm.nih.gov/genbank/, MZ189360; https://www.ncbi.nlm.nih.gov/genbank/, MZ189359; https://www.ncbi.nlm.nih.gov/genbank/, MZ579543; https://www.ncbi.nlm.nih.gov/genbank/, MZ579544; https://www.ncbi.nlm.nih.gov/genbank/, MZ579545.

## Ethics Statement

The animal study was reviewed and approved by the Committee on the Ethics of Animal Experiments of the Harbin Veterinary Research Institute of the Chinese Academy of Agricultural Sciences (CAAS) and the Animal Ethics Committee of Heilongjiang Province, China.

## Author Contributions

SW, FM, and XC conceived the study and designed the experimental procedures. LQ, FM, and SW performed the experiments. LQ, FM, HH, MS, WZ, and SW analyzed the data. HH, Y-BY, Y-DT, GW, MS, WZ, and XC contributed reagents and materials. LQ, FM, and SW wrote the manuscript. All authors contributed to the article and approved the submitted version.

## Funding

This work was supported by the National Natural Science Foundation of China (32002249), the Natural Science Foundation of Heilongjiang Province (QC2018030), and the Local Social Science Project (2018QD0031) of Heilongjiang Province and Heilongjiang pig Modern Agricultural Technology Collaborative Innovation System.

## Conflict of Interest

The authors declare that the research was conducted in the absence of any commercial or financial relationships that could be construed as a potential conflict of interest.

## Publisher's Note

All claims expressed in this article are solely those of the authors and do not necessarily represent those of their affiliated organizations, or those of the publisher, the editors and the reviewers. Any product that may be evaluated in this article, or claim that may be made by its manufacturer, is not guaranteed or endorsed by the publisher.
